# Dextroposition of the Heart From Tension Hydrothorax in a Patient With Malignant Pleural Effusion

**DOI:** 10.7759/cureus.82551

**Published:** 2025-04-18

**Authors:** Julia Y Lu, Emily Fritzmann, David W Hsia, Charles Lanks

**Affiliations:** 1 Internal Medicine, Harbor-UCLA (University of California, Los Angeles) Medical Center, Torrance, USA; 2 Pulmonary and Critical Care Medicine, Harbor-UCLA (University of California, Los Angeles) Medical Center, Torrance, USA

**Keywords:** malignant pleural effusion (mpe), metastatic breast cancer, point-of-care-ultrasound, tension hydrothorax, video-assisted thoracoscopy

## Abstract

Tension hydrothorax, though rare, is a life-threatening complication of malignant pleural effusion (MPE). Early recognition and intervention are crucial to prevent hemodynamic instability and respiratory failure. We present a case of a 68-year-old woman with a history of breast cancer who presented with progressive dyspnea and significant weight loss, in addition to dextroposition of her heart on exam. Imaging revealed a large left-sided pleural effusion with pleural nodularity and displacement of both the heart and great vessels into the right hemithorax, consistent with tension hydrothorax from MPE. Management included immediate pleural drainage with tube thoracostomy, which led to symptomatic relief, and the diagnosis of MPE was later confirmed through pleural biopsy. This case highlights the importance of rapid diagnostic and therapeutic approaches in extreme and life-threatening presentations of MPE.

## Introduction

Malignant pleural effusion (MPE), a common complication in advanced malignancies, occurs in 10-20% of cancer patients and is most commonly seen with breast and lung cancers [1,2]. Although MPE is well described, the progression to tension physiology is rare and often under-recognized [3,4]. MPE is defined by the presence of cancer cells in pleural fluid or tissue, indicating disease dissemination [5,6]. The pathophysiology of MPE is multifactorial, often involving disruptions of lymphatics and local inflammation [1]. While pleural effusions are common in patients with malignancy, tension hydrothorax is a rare but critical clinical entity, which can lead to acute hemodynamic compromise and death [3,4,7].

## Case presentation

A 68-year-old woman with a history of left-sided breast cancer status post left mastectomy six years ago, with subsequent radiation and adjuvant chemotherapy, presented to the emergency department for evaluation of progressive dyspnea and subjective worsening shortness of breath at rest over the past three months that improved with lying flat. Additionally, she had unintentional weight loss of 35 pounds during this time. Otherwise, the patient had no other positive review of systems. Two years before this presentation, she had self-discontinued follow-up for her breast cancer as her symptoms had subjectively resolved. Unfortunately, there was no further information on the nature of her breast malignancy as she received all of her care in a different country prior to presenting for this hospitalization. On examination, she appeared thin and in moderate respiratory distress. Her vital signs included a heart rate of 120 beats per minute, respiratory rate of 20 breaths per minute, blood pressure of 107/67 mmHg, and oxygen saturation of 93% on room air. Physical examination of the chest revealed absent breath sounds over the left hemithorax and a markedly displaced heart sound and point of maximal impulse toward the right chest. A well-healed horizontal incision on her left chest wall was consistent with her prior mastectomy.

**Figure 1:**
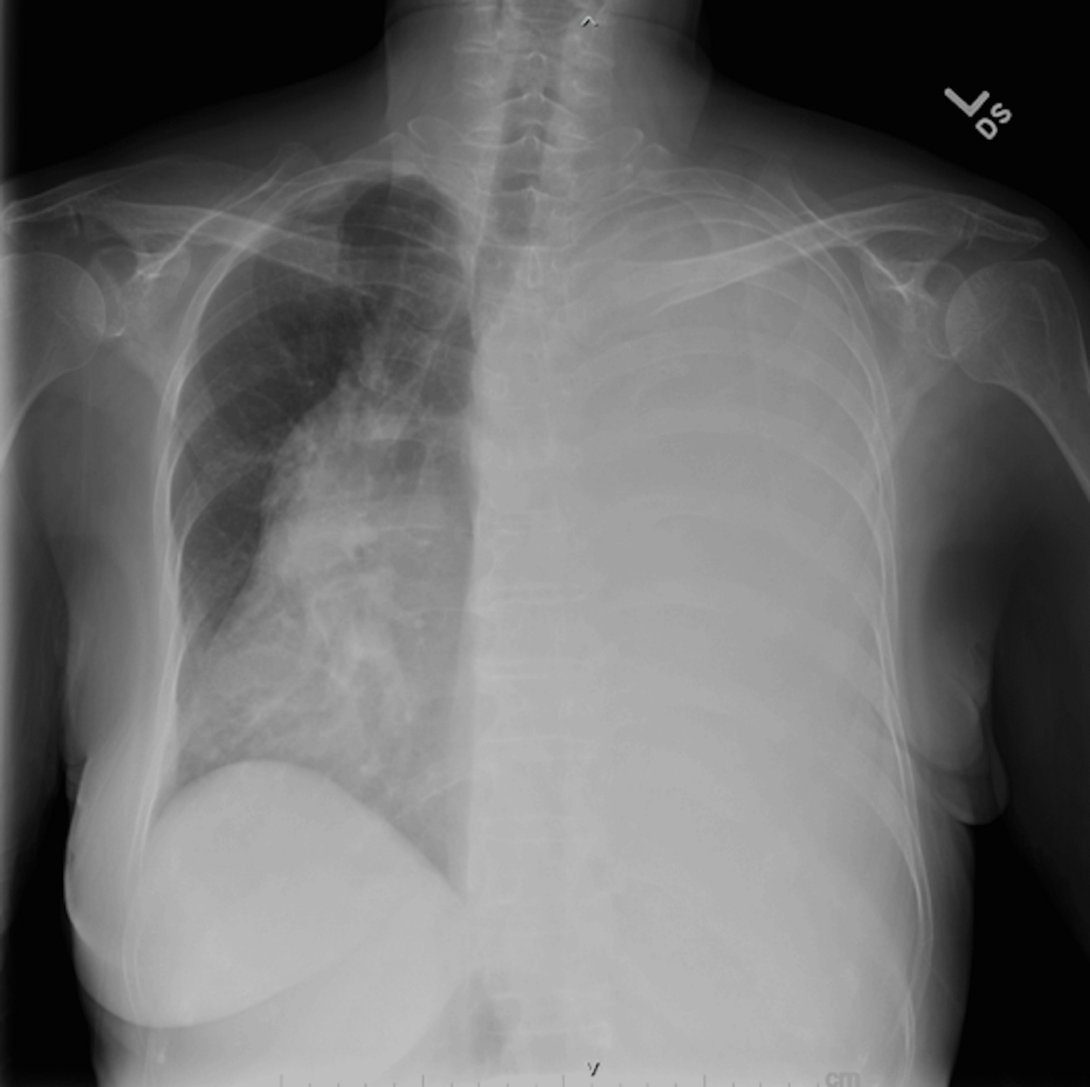
Chest X-ray. Plain radiograph of the chest showing opacification of the left hemithorax, with dextroposition of the heart and mediastinal structures to the right.

**Figure 2:**
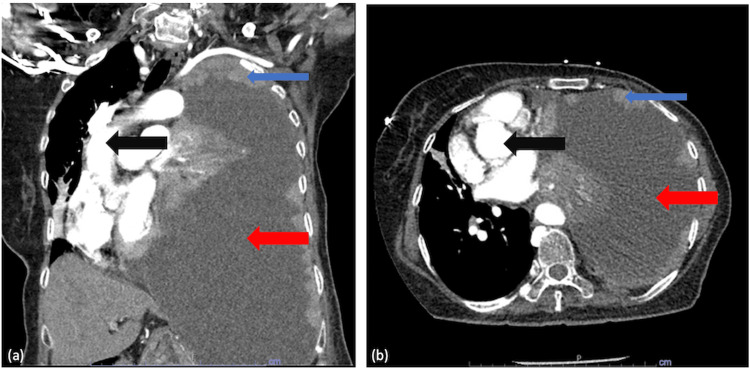
Computed tomography of the chest. (a) Coronal view and (b) axial view. A large pleural effusion (red arrow) with complete atelectasis of the left lung with dextroposition of the heart and mediastinal structures (black arrows) to the right. There is also significant pleural nodularity (blue arrows).

**Figure 3:**
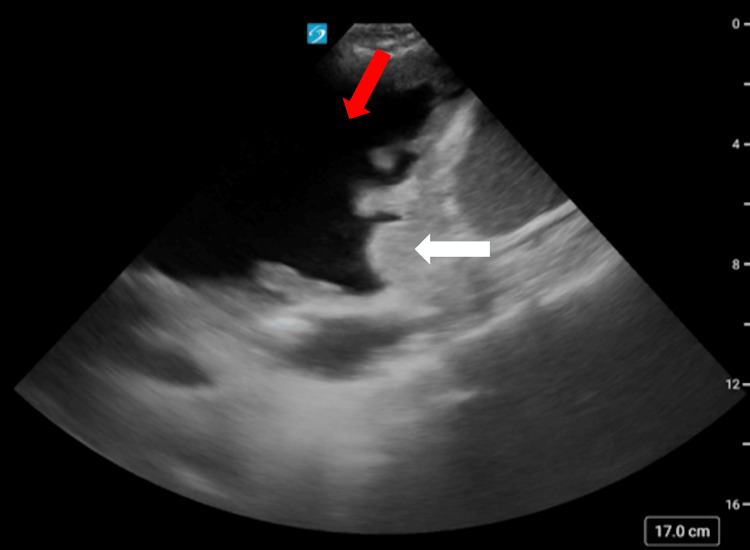
Point-of-care ultrasound. Point-of-care ultrasound of the left chest in the midaxillary line showing left-sided pleural effusion (red arrows) with pleural nodularity (white arrows).

Initial laboratory studies revealed mild leukocytosis with a white blood cell count of 14,000/mm³ and 89.7% neutrophils. Other blood counts, chemistry panels, and B-natriuretic peptide levels were within normal limits. Blood cultures were collected on admission and had been with negative growth. A chest X-ray demonstrated complete opacification of the left hemithorax with a rightward mediastinal shift (Figure 1), and computed tomography (CT) demonstrated large unilateral pleural effusion (Figure 2). Point-of-care ultrasound (POCUS) revealed thickened parietal pleura with multiple nodules, concerning for malignant dissemination (Figure 3). Given her findings, she was diagnosed with tension hydrothorax due to a large pleural effusion that was suspected to be malignant.

**Figure 4:**
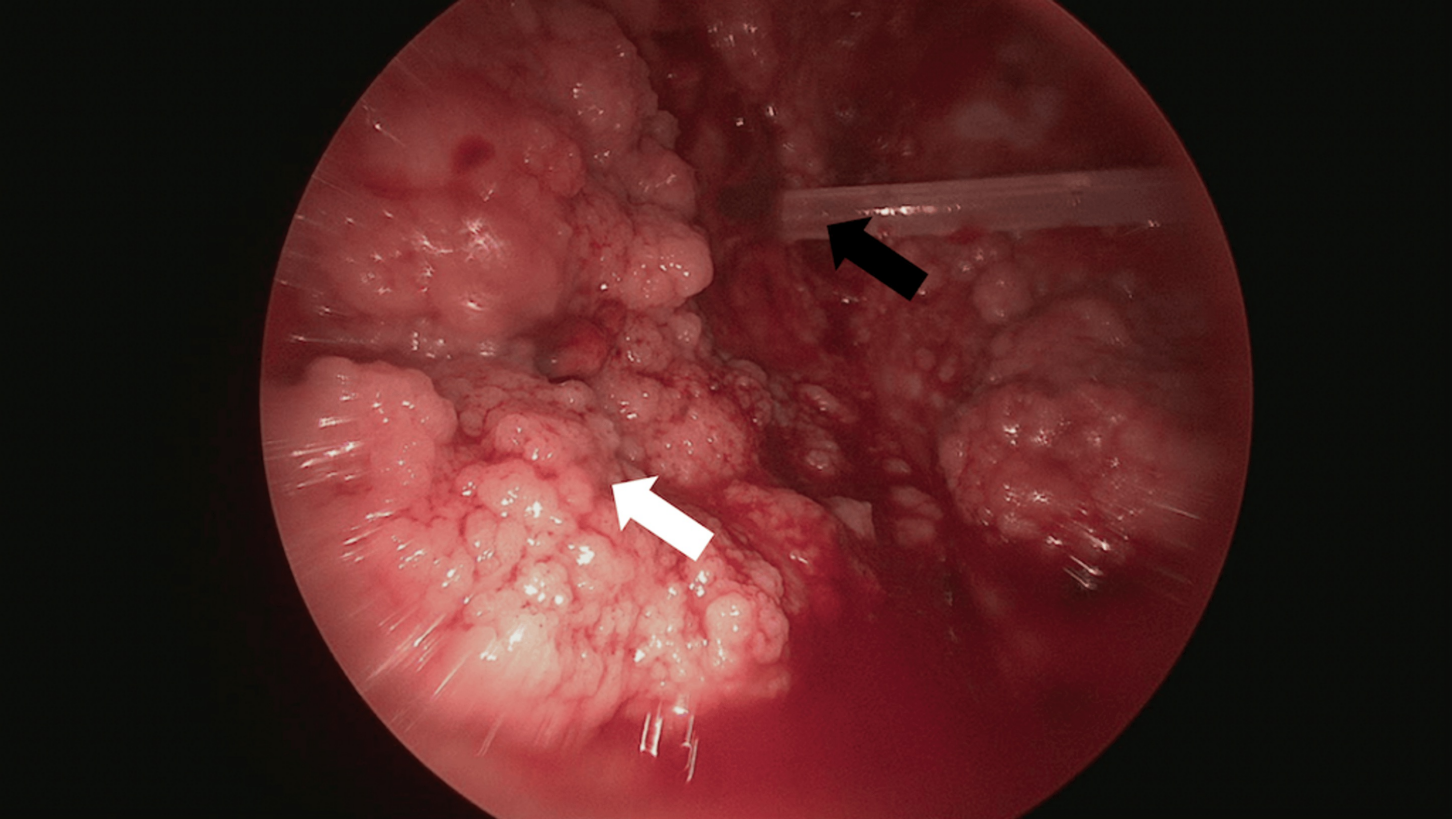
Thoracoscopic view of the left parietal pleural nodules. The indwelling tunneled pleural catheter (black arrow) can be seen within the pleural space surrounded by groups of pleural nodules (white arrow).

Immediate management included placement of a 14 French pigtail catheter for pleural drainage. Over the course of her hospitalization, a total of 15 liters of pleural fluid was removed. Analysis of the fluid revealed a body lactate dehydrogenase level of 390 U/L, consistent with an exudative effusion by Light’s criteria, and negative bacterial, fungal, and acid-fast bacillus cultures. Pleural fluid cytology identified malignant cells consistent with carcinoma. However, despite evaluating over four liters of pleural fluid, malignant cells were insufficient for next-generation sequencing or definitive identification of the primary malignancy, so she underwent thoracoscopic biopsy of her pleural nodules (Figure 4).

**Figure 5:**
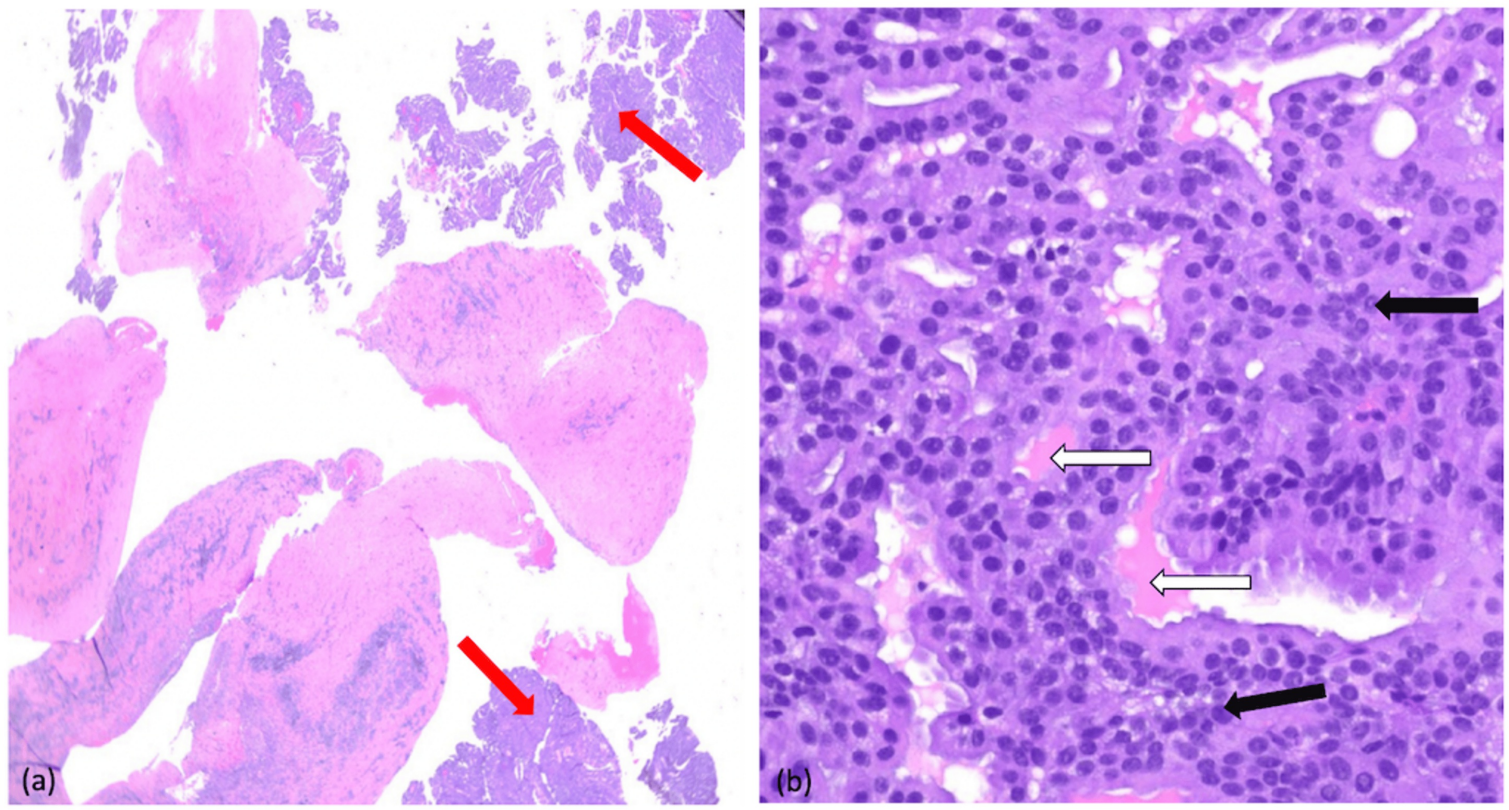
Hematoxylin and eosin stain. (a) Low power view - hematoxylin and eosin stain of the pleural biopsy showing irregular glandular structures (red arrows) embedded within desmoplastic stroma. (b) High power view - hematoxylin and eosin stain of the pleural biopsy showing enlarged, hyperchromatic nuclei with irregular nuclear membranes (black arrow), crowding, and loss of normal architecture, consistent with malignancy. There is also evidence of eosinophilic and mucin secretion (white arrows), suggesting adenocarcinoma differentiation.

**Figure 6:**
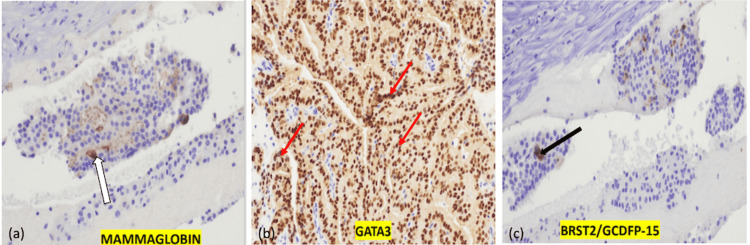
Immunohistochemical stains. (a) Mammaglobin: Positive cytoplasmic staining (white arrow), a specific marker for breast carcinoma. (b) GATA3: Strong nuclear positivity (red arrows), supporting a breast primary origin. (c) BRST2/GCDFP-15: Focal positivity (black arrow), consistent with breast tissue differentiation.

Subsequent stain analysis of the obtained samples confirmed adenocarcinoma (Figure 5). The tumor cells exhibited strong positivity for mammaglobin (Figure 6A), GATA3 (Figure 6B), and BRST2 (Figure 6C), while staining was negative for TTF-1 and napsin A, effectively ruling out a lung primary. To support this, studies have shown that mammaglobin and GATA3 are expressed in 64% and 72% of mammary carcinomas, respectively, whereas TTF-1 and napsin A are positive in 80% and 77% of pulmonary adenocarcinomas, respectively [8]. Therefore, the presence of mammaglobin and GATA3, along with the absence of TTF-1 and napsin A, supports the diagnosis of metastatic breast adenocarcinoma in this patient. The immunohistochemical assay was found to be positive for hormone receptor, with negative HER2 expression.

Due to the patient’s large and continuous output from her chest tube, she elected to undergo tunneled indwelling pleural catheter (TIPC) placement prior to hospital discharge. At a follow-up visit in the outpatient pulmonology clinic, she reported improved dyspnea with continued drainage. However, she had multiple subsequent hospital admissions for nausea and vomiting secondary to her chemotherapy with ribociclib, a cyclin-dependent kinase 4 and 6 inhibitor, primarily used in hormone receptor-positive and HER2-negative breast adenocarcinoma. Four months after TIPC placement, the patient expressed her intent to travel back to her home country with the TIPC in place and was subsequently lost to follow-up.

## Discussion

While tension hydrothorax has been described in case reports since its original description in 1966 by Rabinov et al. [3], its precise incidence remains unknown. Clinically, tension hydrothorax occurs when a large pleural effusion causes hemodynamic compromise as a result of direct mediastinal compression [4,7]. The high intrapleural pressures are due to both increased fluid production and decreased drainage of fluid within the pleural space. This increased pressure causes impaired cardiac diastolic filling and reduced venous return to the heart. Subsequently, diminished cardiac output produces hemodynamic instability and obstructive shock. Importantly, there can be tension physiology with reduced cardiac output even before the development of frank hypotension. Rapid diagnosis and prompt decompression are imperative to prevent further hemodynamic compromise, and delayed recognition may lead to rapidly progressive respiratory failure, cardiovascular collapse, and death [4].

Although pleural effusions may occur in 2% to 11% of patients with breast cancer, caused by dissemination from pleural lymphatics, the incidence of developing a tension hydrothorax as a result of MPE is relatively rare [2]. MPE is defined by the presence of cancer cells in the pleural fluid or tissue, indicating disease dissemination. In patients such as ours, it can be the first and only manifestation of recurrence. The pathophysiology of MPE is multifactorial and often attributed to a disruption of lymphatic vessels, leading to impaired drainage and fluid accumulation; local chemokines increase the permeability of the vascular and pleural membranes, causing fluid dysregulation with subsequent accumulation [1].

Patients with MPE may present with varying degrees of dyspnea, which is dependent on both the volume of pleural fluid and the rate of its accumulation. When MPE progresses to tension hydrothorax, which can be insidious, patients often present with severe respiratory distress and chest pain. These patients may frequently have accompanying hypotension, pulsus paradoxus, tachycardia, and jugular venous distention from increased intrathoracic pressure, with physical examination notable for decreased breath sounds and dullness to percussion on the affected side [4]. Dextroposition of heart sounds may occur in left-sided tension hydrothorax due to rightward mediastinal shift.

The initial evaluation of a pleural effusion is commonly accomplished with a chest X-ray. Effusions as small as 200 mL can cause costophrenic angle blunting on chest X-ray, while even smaller effusions can be detected in the lateral decubitus position [9]. Contrast-enhanced chest CT can show the presence and degree of pleural thickening, detect pleural nodules and lymphadenopathy, and sometimes help to identify the primary neoplasm. Relative to X-ray, CT also allows for more precise quantification of effusion size [5]. Similar to CT imaging, magnetic resonance imaging and positron emission tomography can demonstrate structural pleural abnormalities or potential chest wall involvement, but are limited in their ability to differentiate between benign and malignant causes of pleural effusion [5]. Finally, POCUS is often more immediately accessible than CT. Despite the dependence of sensitivity and specificity on user experience, it can have superior accuracy compared to CT in detecting morphological features associated with MPE, such as diaphragmatic abnormalities, pleural thickening, and visceral nodularity [10].

Thoracentesis is the initial procedure of choice when MPE is suspected and should be performed under ultrasound guidance. Removal of pleural fluid provides both diagnostic information as well as relief from respiratory symptoms if an adequate volume of fluid is drained [11]. MPE is typically exudative but can present as a transudate in 5% to 10% of cases. The initial diagnostic yield for malignancy by pleural fluid cytology is only 40-60% but increases by 30% with a second thoracentesis, and 10% with a third [6]. If clinical suspicion for MPE remains high despite negative pleural fluid cytology, pleural biopsy should be considered. Thoracoscopy allows for direct visualization and sampling of pleural metastases and has a diagnostic sensitivity as high as 98% [6,11].

The management of MPE is typically directed at symptom palliation and is tailored to each individual patient’s goals of care [2]; no specific procedural intervention has been shown to prolong survival [11]. Thoracentesis is often the initial first step that offers rapid symptom relief while simultaneously obtaining fluid for analysis, but does not address fluid re-accumulation. For long-term management, as an alternative to serial thoracenteses, pleurodesis or placement of an indwelling pleural catheter (IPC) should be considered. Pleurodesis may be achieved via video-assisted thoracoscopy [12], which induces inflammation and adhesion of the pleural space by inflicting direct damage to the pleural surfaces via a chemical sclerosant or mechanical abrasion. It can also be achieved simply through placing an IPC, which allows for adhesion formation through local inflammation and gradual lung expansion by reducing pleural effusion, allowing pleural surfaces to adhere [13]. It is worth noting that those with non-expandable lungs or rapidly re-accumulating fluid may not be suitable candidates for pleurodesis. An IPC is generally well tolerated and is an efficient method for outpatient drainage of recurrent MPE [11].

**Figure 7:**
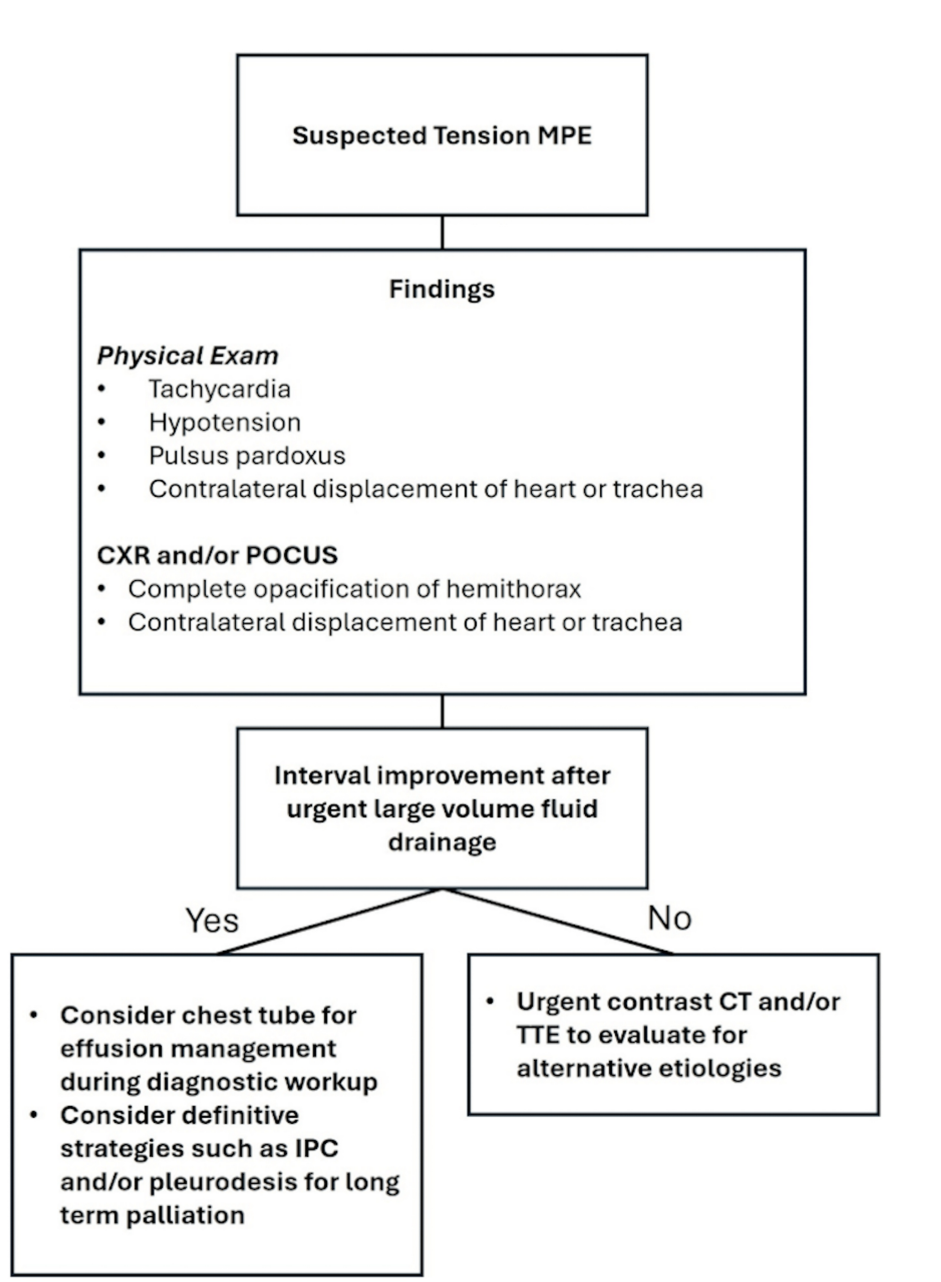
A step-wise approach to tension hydrothorax from MPE. MPE: malignant pleural effusion; CXR: chest X-ray; POCUS: point-of-care ultrasound; IPC: indwelling pleural catheter; TTE: transthoracic echocardiogram.

However, given the rarity of tension physiology in MPE, an algorithm focusing on its early identification may help guide prompt management (Figure 7). Recognizing key signs and symptoms, such as mediastinal shift, hypotension, pulsus paradoxus, tachycardia, and jugular venous distension, is critical for prompt diagnosis [4]. Furthermore, reassessment after large-volume drainage is necessary to ensure interval improvement, as persistent hemodynamic compromise may indicate the need for tube thoracostomy over serial thoracenteses [3]. Beyond immediate fluid removal, management should include a lower threshold for definitive palliative interventions such as IPC placement or pleurodesis to mitigate the risk of life-threatening recurrence. Although these considerations lack robust clinical evidence, they provide a framework for clinicians managing tension hydrothorax, emphasizing both acute stabilization and long-term palliation. Since tension hydrothorax involves large volume effusions, complications from chest tube placement and rapid drainage may occur and include patient discomfort from negative pressure in the pleural space, re-expansion pulmonary edema, and iatrogenic pleural infection if the tube remains in place for an extended period of time.

The finding of MPE is often indicative of disseminated malignancy; thus, these patients tend to have poor prognoses. Median survival after diagnosis ranges from three to 12 months and depends on the underlying malignancy type, Eastern Cooperative Oncology Group (ECOG) score, stage of malignancy at diagnosis, and treatment options available [14]. The LENT prognostic score uses pleural fluid lactate dehydrogenase (LDH), ECOG score, serum neutrophil-to-lymphocyte ratio, and tumor type to predict survival in people with MPE. In one study, those with low-risk, moderate-risk, and high-risk LENT scores had median survival of 319 days, 130 days, and 44 days, respectively [14]. Prognosis related to malignant tension hydrothorax has not been well defined, but after initial drainage, it is likely to be similar to that for MPE alone.

## Conclusions

This case highlights the importance of early recognition and management of MPE. Significant displacement of intrathoracic structures can sometimes predate hemodynamic compromise, and rapid intervention can be life-saving. The use of POCUS can now assist in diagnosis and therapeutic intervention in addition to preexisting image modalities. Even though there are many options to provide symptomatic relief of MPE, long-term management should be tailored to the patient’s goals of care, and multidisciplinary collaboration is essential for optimizing outcomes in this challenging clinical scenario.
